# Endoscopic Mitral Surgery in Noonan Syndrome—Case Report and Considerations

**DOI:** 10.3390/jcm14020583

**Published:** 2025-01-17

**Authors:** Marius Mihai Harpa, Emanuel-David Anitei, Claudiu Ghiragosian, Paul Calburean, Diana Roxana Opris, Marian Cosmin Banceu, Emil Marian Arbanasi, Horatiu Suciu, Hussam Al Hussein

**Affiliations:** 1 Department of Surgery IV, George Emil Palade University of Medicine, Pharmacy, Science and Technology of Targu Mures, 38 Gheorghe Marinescu Street, 540139 Targu Mures, Romania; marius.harpa@umfst.ro (M.M.H.); claudiu.ghiragosian@umfst.ro (C.G.); calbureanpaul@gmail.com (P.C.); dianaroxana.opris@yahoo.com (D.R.O.); cosmin.banceu@umfst.ro (M.C.B.); emil.arbanasi@umfst.ro (E.M.A.); horisuciu@gmail.com (H.S.); alhussein.hussam@yahoo.com (H.A.H.); 2 Department of Cardiovascular Surgery, Emergency Institute for Cardiovascular Diseases and Transplantation Targu Mures, 50 Gheorghe Marinescu Street, 540136 Targu Mures, Romania

**Keywords:** endoscopic cardiac surgery, mitral surgery, Noonan syndrome, pectus excavatum, minimally invasive

## Abstract

**Background**: Totally endoscopic techniques have become increasingly popular in cardiac surgery, with minimally invasive mitral valve repair emerging as an effective alternative to median sternotomy. This approach could be particularly advantageous for patients with Noonan syndrome, who often present with structural thoracic anomalies and other comorbidities like bleeding disorders. Endoscopic mitral valve surgery is rapidly establishing itself as the new standard of care for mitral valve operations, demonstrating both safety and efficacy. Noonan syndrome is an autosomal-dominant multisystem disorder with variable expression and is the second most common syndromic cause of congenital heart disease, surpassed only by Down syndrome. A wide spectrum of cardiovascular phenotypes is associated with Noonan syndrome, including pulmonary valve stenosis (often with dysplastic valves), hypertrophic cardiomyopathy, secundum atrial septal defect and mitral valve abnormalities. **Methods**: Given the limited data in the literature regarding the experience of other centers with endoscopic mitral surgery in patients with this condition, we aim to present the case of a 46-year-old male with a known diagnosis of Noonan syndrome who presented to a cardiologist with a 6-month history of dyspnea and fatigue. Transthoracic echocardiography revealed severe mitral regurgitation. Following multidisciplinary discussions within the Heart Team and after obtaining informed consent from the patient and his family, the decision was made to proceed with totally endoscopic mitral valve repair. **Results**: The patient experienced an uneventful postoperative course and was discharged 8 days after the procedure. In this case, endoscopic surgery was essential for successfully repairing the mitral valve. Structural abnormalities, such as chest wall deformities causing heart malrotation and atypical positioning, significantly impaired visualization. **Conclusions**: The endoscopic approach provided superior access to the mitral valve, enabling precise and effective repair. Additionally, it offered benefits such as improved esthetic outcomes, faster recovery, and a reduced risk of exacerbating thoracic deformities due to improper sternal bone healing.

## 1. Introduction

Patients with genetic syndromes that include uncorrected sternal anomalies present a significant challenge in cardiac surgery, particularly in endoscopic cardiac procedures. Current minimally invasive techniques offer several advantages, including superior cosmetic outcomes, shorter hospital stays, reduced pain, and fewer reinterventions for hemorrhage [1,2]. Noonan syndrome (NS), first described in 1963, is among the most common causes of syndromic congenital heart disease, second only to Trisomy 21. The condition is genetically heterogeneous, with an estimated prevalence of 1 in 1000 to 1 in 2500 live births [3,4]. NS is characterized by a variety of features, including distinctive facial traits (hypertelorism, ptosis, and low-set ears), short stature, developmental delay of varying severity, lymphatic vessel dysplasia, bleeding disorders, cardiac defects, and chest abnormalities (pectus excavatum, carinatum). Mutations in the RAS-MAPK signaling pathway, particularly gain-of-function mutations, are found in up to 60% of patients with NS. Although most cases of Noonan syndrome are inherited in an autosomal-dominant manner, a recessive form has recently been linked to biallelic variants in the LZTR1 gene [5,6]. The combination of pectus excavatum and mitral valve repair in patients with Noonan syndrome is exceedingly rare due to the unique interplay of congenital structural anomalies in this population. However, the coexistence of pectus excavatum requiring simultaneous consideration during mitral valve repair presents a unique surgical challenge, with only one similar case reported in the literature to our knowledge, describing a patient with Noonan syndrome, skeletal anomalies, and mitral valve repair through a minimally invasive approach. Over 80% of patients diagnosed with Noonan syndrome exhibit cardiac anomalies, with 6% presenting mitral valve abnormalities. Mitral valve pathology is particularly common among individuals with mutations in the PTPN11 and RAF1 genes [5,6,7]. Approximately 23% of patients with Noonan syndrome present with chest abnormalities, which could be a contraindication for endoscopic cardiac surgery [8,9]. The Haller index, a measurement of the ratio between the transverse diameter and the anteroposterior diameter of the chest, is critical for assessing the severity of pectus excavatum and guiding surgical decisions. An index greater than 3.25 is typically considered to be indicative of severe deformity warranting surgical correction. In cases involving cardiac dysfunction, such as mitral valve disease, elevated Haller index values may further influence the approach by necessitating tailored surgical strategies to optimize both thoracic geometry and cardiac repair outcomes. Another important parameter is the correction index, which measures the percentage of chest compression, with values exceeding 28% often used to identify cases requiring surgical intervention [10,11,12,13,14]. We present the case of a 46-year-old male with Noonan syndrome who complained of exertional dyspnea and was admitted to our clinic, where he was diagnosed with severe mitral regurgitation due to anterior mitral valve (AML) prolapse with A2 segment flail. The patient also presented with significant pectus excavatum and mild thrombocytopenia. Coronary angiography revealed a co-dominant coronary system, while CT angiography of the thoraco-abdomino-pelvic region showed no vascular calcifications or anomalies. After preoperative assessment and discussion within the Heart Team, the patient underwent endoscopic mitral valve repair, with an uneventful postoperative course and favorable outcome.

## 2. Case Report

**Figure 1:**
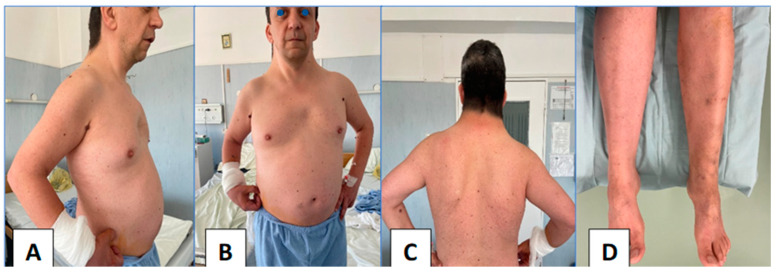
Pictures published with the patient’s consent. (**A**) Lateral view, pectus excavatum. (**B**) Hypertelorism, palpebral ptosis, micrognathia, prominent nasolabial folds, low-set ears, pterygium colli. (**C**) Low posterior hairline. (**D**) Lymphedema of the lower extremities.

**Figure 2:**
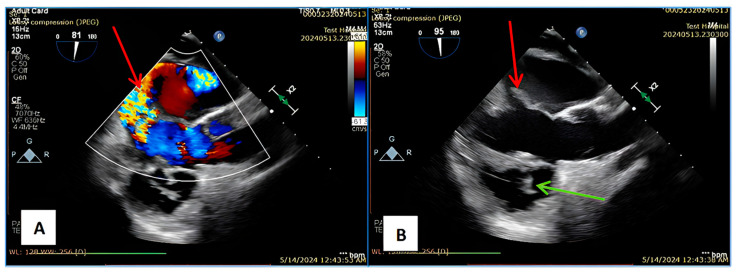
(**A**) TEE—severe regurgitation with an eccentric jet directed posterior laterally, exhibiting a Coanda effect. (**B**) TEE—red arrow: mitral valve prolapsed, with a myxomatous appearance, elongated and flailing due to chordal rupture at the A2–A3 level; green arrow: tricuspid valve.

**Figure 3:**
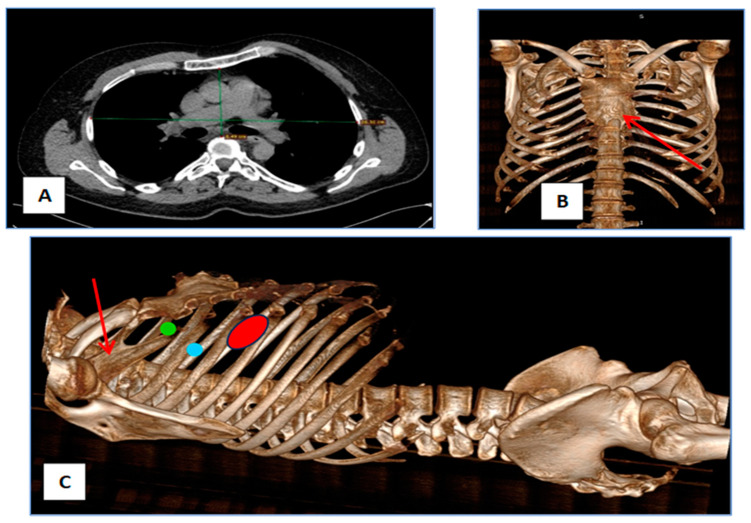
(**A**) CT scan demonstrating pectus excavatum with a Haller index of 3.1. (**B**) CT scan and 3D bone reconstruction: the red arrow denotes a malformation of the upper sternum. (**C**) Lateral view: the red arrow highlights the fusion of the first and second ribs. The red dot marks the incision site in the fourth intercostal space, the green dot indicates the position of the 3D camera, and the blue dot marks the location of the Chitwood clamp.

**Figure 4:**
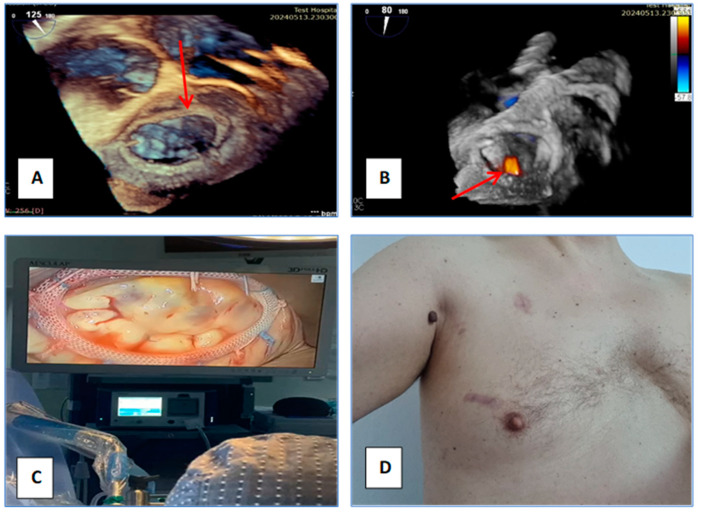
(**A**) TEE—3D imaging, visible annuloplasty ring. (**B**) TEE—red arrow indicates a mild regurgitation jet at A2 level. (**C**) Postoperative aspect of the mitral valve. (**D**) Appearance of the scar 6 months postoperatively.

**Figure 5:**
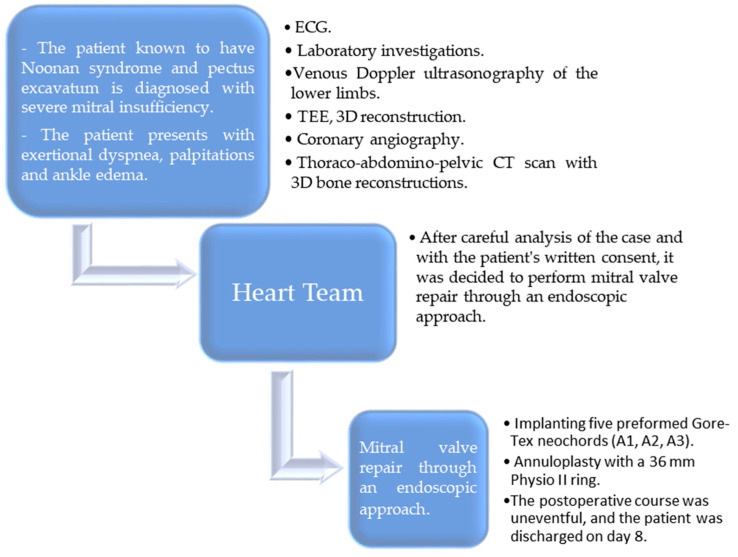
Case timeline. ECG—electrocardiogram; TEE—transesophageal echocardiography.

We report the case of a 48-year-old male with a confirmed diagnosis of Noonan syndrome, depressive disorder, and chronic alcohol abuse, presenting with exertional dyspnea, episodic palpitations, and bilateral lower limb edema. The patient reported no known family history of Noonan syndrome. On clinical examination, he demonstrated normal cognitive function, a height of 1.64 m, and phenotypic hallmarks of Noonan syndrome, including hypertelorism, palpebral ptosis, pterygium colli, low-set ears, a low posterior hairline, micrognathia, prominent nasolabial folds, pectus excavatum, and lymphedema of the lower extremities (Figure 1A–D). Preoperative transesophageal echocardiography (TEE) revealed a horizontalized heart requiring modified imaging views. The left ventricle was non-dilated, mildly hypertrophic with a basal interventricular septum (IVS) of 15 mm, with preserved contractility and no systolic anterior movement (SAM). The right ventricle was non-dilated and efficient, while the left atrium was moderately dilated. The mitral valve showed a retracted posterior mitral leaflet (PML) and a myxomatous AML with flail at A2–A3 due to chordal rupture, causing severe regurgitation with a postero-lateral eccentric jet and Coanda effect. The tricuspid valve exhibited mild regurgitation (Figure 2A,B). Coronary angiography identified a co-dominant coronary circulation without significant stenotic lesions. Thoraco-abdomino-pelvic computed tomography angiography revealed no vascular malformations or calcifications. Thoracic imaging demonstrated pectus excavatum with a correction index (CI) of 24%, Haller index (HI) of 3.1 and fusion of the first two ribs on the right hemithorax (Figure 3A–C). Venous Doppler ultrasonography of the lower limbs excluded significant venous pathology. The patient’s electrocardiogram (ECG) demonstrated signs of atrial flutter with a ventricular rate of 98 beats per minute. Laboratory investigations revealed mild thrombocytopenia and an extended activated partial thromboplastin time (aPTT) of 36 s (reference range: 21–29 s). No history of spontaneous bleeding was documented. The surgical intervention was conducted utilizing a fully 3D endoscopic system (Aesculap Einstein vision, 3.0 FI, Tuttlingen, Germany) incorporating CO_2_ insufflation, a 3D videoscope with 0° or 30° angles, a pneumatic holding arm (B. Braun, Unitrac, Melsungen, Germany), soft tissue retractor (Geister, ValveGate, Tuttlingen, Germany) and Estech Atrial Lift System (Atricure, Mason, OH, USA). Cardiopulmonary bypass was established via cannulation of the right internal jugular vein, right femoral vein, and right femoral artery. A fourth intercostal space incision along the anterior axillary line was employed for instrument access, and a first intercostal space port was used for the videoscope. An aortic Chitwood clamp (Geister, Tuttlingen, Germany) was utilized through the third intercostal space. The mitral valve was repaired by implanting five preformed Gore-Tex neochords (Seramon chordae loop, Serag Wiessner, Naila, Germany) at segments A1, A2, and A3, complemented by annuloplasty with a 36 mm Physio II mitral ring (Edwards Lifesciences LLC, Irvine, CA, USA). Postoperative findings (TEE) indicated that mitral valve repair and annuloplasty were successfully performed. Three-dimensional imaging confirmed accurate positioning, with minimal residual regurgitation at A2, which was deemed clinically insignificant (Figure 4A–D). The cardiopulmonary bypass duration was 147 min, with an aortic cross-clamp time of 98 min. The patient was extubated six hours postoperatively, exhibited an uncomplicated recovery trajectory, and was discharged on postoperative day 8. We added a case timeline to facilitate a clearer overview of the patient’s perioperative course (Figure 5).

## 3. Discussion

Noonan syndrome, first described in 1963 by Jacqueline Noonan, is a relatively common multisystem disorder predominantly inherited in an autosomal-dominant pattern, though autosomal-recessive inheritance linked to LZTR1 mutations has also been reported [10,11]. NS is characterized by several hallmark features, including craniofacial anomalies such as ptosis (16%), hypertelorism (25%), and low-set ears (16%); short stature; variable developmental delay; lymphatic dysplasia; bleeding diatheses; and chest wall abnormalities, most notably pectus excavatum. Cardiac anomalies are a defining feature, with pulmonary stenosis (often with dysplastic valves) occurring in 50–60% of cases, hypertrophic cardiomyopathy in 20%, secundum atrial septal defects in 6–10%, and these conditions are often associated with mitral valve abnormalities [3,12]. Some studies report that 80–90% of patients diagnosed with Noonan syndrome present with cardiac abnormalities, with 6% exhibiting mitral valve defects. These findings are consistent with those of Léa Linglart et al. in their study “Cardiovascular Abnormalities and Gene Mutations in Children with Noonan Syndrome” [10] which noted mitral regurgitation in 16–27% of cases. Pectus excavatum is a relatively common condition that can occur as an isolated pathology. However, in 23% of cases, it is associated with other characteristic features of Noonan syndrome. Some studies report this prevalence to be as high as 31% [9,10,13]. The surgical indication for pectus excavatum is based on a correction index (CI) exceeding 28% and a Haller index (HI) greater than 3.25. These parameters suggest the need for correction using various techniques, either conventional or minimally invasive, with the Nuss procedure being the most well-known approach [14]. Our patient presented with a correction index (CI) of 24% and a Haller index (HI) of 3.1, classifying as moderate pectus excavatum. The spirometry results were within normal limits. A sternotomy was deemed unsuitable due to the horizontalized and significantly left-deviated cardiac position, which would have resulted in suboptimal exposure of the mitral valve. Additionally, the asymmetric thoracic structure and altered sternum shape posed a risk of pathological healing, potentially exacerbating the pectus excavatum deformity. Sternotomy could also have prolonged physical recovery and increased the risk of complications such as sternal dehiscence or pseudoarthrosis, potentially necessitating reoperation. Thoracic anomalies or sternal deformities can represent a contraindication to endoscopic cardiac surgery. Some studies classify thoracic deformities as relative contraindications for minimally invasive or endoscopic cardiac surgery, and even absolute contraindications in cases of severe anomalies. However, in the hands of an experienced surgeon, supported by a skilled and well-prepared team with a carefully established operative plan, endoscopic surgery can result in successful outcomes [8,9,15]. In patients without diagnosed genetic syndromes or significant structural abnormalities of the thoracic cage, endoscopic cardiac surgery has proven to be highly effective. It offers superior cosmetic results, increased patient satisfaction, reduced postoperative complications, and shorter hospital stays [16,17]. In patients with genetic syndromes that include uncorrected congenital sternal abnormalities, there are a lack of extensive studies evaluating the feasibility and safety of endoscopic cardiac surgery. However, the study by Johan van der Merwe et al. [18] provides compelling evidence that sternal deformities are not contraindications for endoscopic cardiac procedures. In his mini-series involving seven patients with Noonan syndrome and uncorrected thoracic deformities who underwent mitral valve repair via an endoscopic approach, he demonstrates that endoscopic surgery can also be performed in patients with severe pectus excavatum. Two of these patients had a Haller index greater than 3.25 and a correction index exceeding 30%, indicating severe pectus excavatum. At 6-month follow-up, none of the patients required reoperation, no residual mitral regurgitation greater than mild was detected, and all patients were classified as NYHA Class I. Moreover, the authors suggest that the benefits of these techniques might even surpass those of conventional approaches in such cases [18,19]. For patients with cardiac pathologies and sternal abnormalities requiring surgical correction, we recommend referral to multidisciplinary centers with extensive experience. Ideally, these centers should either have mixed teams of cardiac and thoracic surgeons or cardiothoracic surgeons who are capable of addressing both cardiac and thoracic conditions in a single surgical session. The endoscopic approach may be superior to conventional sternotomy; however, a direct comparison is not possible due to the lack of studies with significant cohorts of patients diagnosed with Noonan syndrome, severe mitral insufficiency, and pectus excavatum. Nonetheless, the literature does describe studies involving patients with severe mitral insufficiency and structural thoracic anomalies, such as pectus excavatum, who underwent mitral valve repair using minimally invasive or endoscopic approaches. Hemmati Pouya et al. [20] reported on a cohort of 29 patients with Noonan syndrome and cardiac pathology, 62% of whom were male. All patients underwent sternotomy, with seven receiving mitral valve replacement or repair as a concomitant procedure. Notably, 41% of the patients were under 18 years of age, and nearly half had a history of previous sternotomy. The data also revealed that one patient required reoperation for sternal dehiscence, while six patients required prolonged mechanical ventilation due to respiratory failure or pneumonia.

There are just a few published reports describing minimally invasive procedures for patients with Noonan syndrome, including cases where simultaneous correction of thoracic deformities and cardiac pathologies was performed. It should be acknowledged that in some cases with severe deformities, surgical access may be technically impossible. However, recent studies describe the concomitant management of cardiac pathology and pectus excavatum correction, which should be performed by experienced surgeons after a careful assessment of the risk–benefit. However, further data are needed to establish standardized protocols and optimize outcomes for such complex cases [13,21,22,23,24]. The approach for our patient was particularly challenging due to the thoracic anatomy and rigidity of the chest wall. Following consultations with radiologists, we opted to gain access through the fourth intercostal space. The skin incision was aligned with the fifth rib to allow a potential shift to the fifth intercostal space without additional skin incisions if the fourth intercostal approach proved insufficient for mitral valve exposure. A notable challenge arose when it came to positioning the 3D camera due to the fusion of the first two ribs. The camera was inserted through the first intercostal space. Initially, a 0-degree camera was used until the left atrium was opened. For improved visualization of the mitral valve, a 30-degree camera was employed, which provided adequate visualization. To raise the atrial wall, we used a fixed system from Estech, Atricure. Given that the heart is deviated to the left and horizontalized, we believe it would have been beneficial and much more useful to use a type of atrial retractor that allows for different angulations and expands its surface for better mitral valve exposure, as seen with newer-generation retractors. In cases where the heart is severely shifted to the left, the atrial retractor can be placed parasternally on the left side to facilitate visualization. Aortic clamping through the third intercostal space was achieved using a Chitwood clamp, which was feasible due to the absence of calcifications in the ascending aorta and the favorable distance between it and the thoracic wall. This type of atrial lift system and the Chitwood clamp are part of our standard setup for endoscopic surgery. Other methods, such as endoaortic clamping or different types of aortic clamping instruments, might be useful. The papillary muscles did not exhibit any abnormalities. The implanted cords were preformed Goretex cords, and the mitral valve annuloplasty was performed using an Edwards Lifesciences Physio II ring (36 mm). Cardiopulmonary bypass was performed using a standard peripheral technique for minimally invasive cardiac surgery, with venous cannulation via the right femoral and jugular veins, arterial cannulation via the right femoral artery under echocardiographic guidance, and a reperfusion cannula placed in the right lower extremity. Endoscopic cardiac procedures tend to be more time-consuming than traditional approaches, with a higher level of complexity and a longer learning curve [25]. In this particular case, the procedure was more challenging due to the difficult approach, resulting in a total cardiopulmonary bypass time of 147 min and an ischemic time of 98 min. Due to the anatomical alterations in the neck, locating the jugular vein for cannulation and central venous catheter placement can be challenging; therefore, the use of echocardiography was beneficial in guiding the procedure. Transesophageal echocardiography remains the gold standard for evaluating the mitral valve [26]. In this case, due to the horizontalization of the heart, modified imaging planes were necessary for the evaluation of the mitral valve. Preoperative transesophageal echocardiography revealed a horizontally positioned heart with modified and adapted standard imaging views. The left ventricle was non-dilated, mildly hypertrophic, with preserved contractility and a basal interventricular septal thickness of 15 mm, without systolic anterior motion. The right ventricle was non-dilated and functionally adequate, while the left atrium was moderately dilated. The mitral valve showed a retracted posterior mitral leaflet (PML) and a myxomatous anterior mitral leaflet, elongated with flail due to chordal rupture at the A2–A3 level, resulting in severe mitral regurgitation with an eccentric jet directed posterolaterally, demonstrating a coanda effect. The mitral valve was optimally visualized in a modified 90-degree two-chamber view, with the horizontally positioned left ventricle in the foreground, transesophageal echocardiography in the primary plane, and the aortic valve, along with the tricuspid valve visible in the anterior part of the image, with the latter showing mild regurgitation (Figure 2A,B). Postoperatively, the patient is free from significant mitral regurgitation. At the 6-month follow-up, there is still no significant regurgitation, and the incision site has a satisfactory cosmetic appearance (Figure 4A–D). Patients with Noonan syndrome are prone to bleeding diatheses and pulmonary complications due to chest wall deformities, and this risk must be carefully considered during medical management. However, the patient in question does not have a history of spontaneous bleeding, and his respiratory function has been normal. It is known that 50% of individuals with Noonan syndrome exhibit platelet dysfunction, and in addition to this, deficiencies in coagulation factors, such as factor VIII (von Willebrand factor), are also commonly identified. These coagulation abnormalities increase the potential for bleeding complications and must be monitored closely in patients undergoing surgical or invasive procedures [27,28,29]. Preoperative laboratory investigations revealed mild thrombocytopenia with a platelet count of 128,000/µL and an extended activated partial thromboplastin time (aPTT) of 36 s (reference range: 21–29 s). The patient did not experience significant bleeding from the chest drainage tube. Immediately postoperatively, the patient received red blood cell and platelet transfusions, specifically 1 unit of packed red blood cells, 4 units of platelets, and 4 units of plasma. The postoperative course was favorable, with the chest tube being removed on day 3. Lymphatic abnormalities, most commonly peripheral lymphedema, are present in less than 20% of individuals with Noonan syndrome but can cause substantial morbidity. Less commonly reported are hydrops fetalis, pulmonary, testicular, or intestinal lymphangiectasia. Patients with Noonan syndrome are predisposed to chylous effusions and hypoplastic lymphatic vessels. Edema in the lower limbs is most often caused by abnormalities of the lymphatic vessels. To confirm the diagnosis, lymphoscintigraphy or an MRI with T2-weighted sequences could be performed, but these investigations were not necessary in this case. Through clinical diagnosis and differential diagnosis, chronic venous insufficiency was excluded via Doppler ultrasound, renal function was within normal limits, and the patient did not present with advanced heart failure. Typically, the treatment for lymphedema includes physiotherapy, compression therapy, exercise, and manual lymphatic drainage [3,30]. The individual variability of patients with Noonan syndrome makes it challenging to standardize an endoscopic surgical technique. The feasibility of minimally invasive access and the selection of the intercostal space for approach are determined based on preoperative imaging to ensure optimal visualization of the mitral valve. These cases are highly diverse and may include patients with mitral insufficiency and severe thoracic deformities, which can challenge even the most experienced surgeons. However, structural thoracic anomalies should not deter surgeons from providing patients with the full benefits of endoscopic surgery.

## 4. Conclusions

This case presentation highlights that the endoscopic approach can be a safe alternative for patients with chest wall deformities, such as those observed in individuals with Noonan syndrome. However, there are insufficient data and documented cases of endoscopic surgery in patients with Noonan syndrome, making it challenging to standardize an access method due to the clinical variability associated with genetic syndromes that alter the geometry of the chest wall.

The involvement of an experienced multidisciplinary team is essential, as these cases require a personalized approach. This case emphasizes the clinical importance of such teams and demonstrates that the endoscopic approach is a safe method for addressing complex anatomical cases. Further studies and the exploration of alternative surgical techniques and access points are necessary to develop standardized approaches for managing these challenging cases.

## Data Availability

Data Availability Statements are available in the Emergency Institute for Cardiovascular Diseases and Transplantation Targu Mures database and can be requested from corresponding author.
